# Interactions between neurotrophins, mood, and physical activity under the conditions of sleep deprivation

**DOI:** 10.1038/s41398-024-02871-6

**Published:** 2024-03-22

**Authors:** Marcin Sochal, Marta Ditmer, Agata Binienda, Aleksandra Tarasiuk, Piotr Białasiewicz, Szymon Turkiewicz, Filip Franciszek Karuga, Fichna Jakub, Agata Gabryelska

**Affiliations:** 1 Department of Sleep Medicine and Metabolic Disorders, Medical University of Lodz, Lodz, Poland; 2 Department of Biochemistry, Medical University of Lodz, Lodz, Poland

**Keywords:** Scientific community, Clinical genetics

## Abstract

Sleep deprivation (DS) is the forced elimination of sleep. While brain-derived neurotrophic factor (BDNF) has been extensively studied in the context of in mood changes following DS, the role of other neurotrophins remains elusive. This study explores the impact of DS on BDNF, glial cell line-derived neurotrophic factor (GDNF), neurotrophin-3 (NT3), and neurotrophin-4 (NT4) at mRNA and protein level, considering their potential links to mood disturbances. The study involved 81 participants subjected to polysomnography (PSG) and DS. Blood samples, mood assessments, and actigraphy data were collected twice, after PSG and DS. NT mRNA expression and serum protein concentrations of BDNF, GDNF, NT3, and NT4 were measured. Participants were divided into Responders and Non-Responders based on mood improvement after DS. DS reduced BDNF mRNA expression in all participants, with no change in serum BDNF protein. GDNF protein decreased in Non-Responders, while Responders exhibited reduced GDNF mRNA. NT3 protein increased in both groups, while NT3 mRNA decreased in Respondents. NT4 protein rose universally post-DS, but NT4 mRNA remained unchanged. Physical activity (PA) negatively correlated with mRNA expression of BDNF, GDNF, and NT3 post-DS. The study’s short DS duration and exclusion of immature NT forms limit comprehensive insights. GDNF, together with NT3, might play an important role in mood response to DS. PA during DS seems to impair the mRNA expression of NTs in leukocytes. Future studies on the subject of sleep deprivation might consider investigating the relationship between BDNF and NT4 in the context of their apparent redundancy.

## Introduction

Neurotrophins (NT) are proteins necessary to maintain the proper functioning of the central nervous system (CNS), mediating processes like synaptic plasticity and neuro- and gliogenesis. There are four NTs with similar tertiary structures: nerve growth factor (NGF), brain-derived neurotrophic factor (BDNF), neurotrophin-3 (NT-3), and neurotrophin-4 (NT-4). Glial cell line-derived neurotrophic factor (GDNF) has a different structure than the previous four but fulfills similar functions as a neurotrophic factor.

Studies suggest that NTs might be linked to a wide range of psychiatric disorders, including certain sleep disorders. Projects conducted over the recent decades tended to focus on the BDNF, as the most abundant NT in the CNS, which is also involved in memory consolidation and learning through the induction of morphological changes in neurons [1, 2]. Since this NT might cross the blood–brain barrier in both directions, it could be assumed that its levels in the periphery reflect those within the CNS [3]. It has been demonstrated that insomnia and low sleep quality reduce the level of BDNF, which in turn is also altered in related affective disorders like depression [4, 5]. Similar findings were published for GDNF, but neither NT3 nor NT4 were investigated [6, 7].

On the other hand, deprivation of sleep (DS) might promote the production of NTs, which was connected to its anti-depressant effect, frequently observed in individuals with depression. Zucconi et al. demonstrated an upregulated rate of hippocampal neurogenesis after DS [8]. Eckert et al. reported an increase in serum BDNF following partial (<36 hours) DS [9]. Giese et al. made similar findings [10]. What’s important, in their study, depressed subjects who experienced mood improvement in the wake of sleep loss had a higher BDNF level than non-responders, which corroborates the hypothesis that this NT conveys the therapeutic effects of DS [10].

However, little is known about other mechanisms underlying the response to sleep loss, including the contribution of other NTs. The relationship between GDNF and depression suggests that it might act in parallel to BDNF. However, this has not been investigated up to date [11]. The only available project on the subject was conducted on a small number of participants and did not include mood evaluation [12]. Moreover, proteins usually were not evaluated in tandem with their mRNA expression. This aspect is particularly important, as the process of their synthesis involves biologically active immature pre-peptides, having their own receptor—p75NTR. Measuring both mRNA and protein would give more insight into processes occurring under the conditions of acute sleep loss.

Physical activity (PA) was shown to significantly affect the production of different NTs. However, this aspect was overlooked in studies on DS [13, 14].

Studies on subjects without affective disorders are also lacking as usually depressed subjects were involved, who were not naive to pharmacological intervention, which could skew the results—BDNF and GDNF were shown to be affected by antidepressants, albeit not unanimously [7, 10, 15].

Thus, the present study aims to compare the levels of mRNA expression and serum protein concentration of BDNF, GDNF, NT3, and NT4 after a night of normal sleep and DS, depending on changes in the severity of depressive symptoms as well as evaluate correlations between mentioned NTs, various subjective sleep parameters, and physical activity.

## Materials and methods

### Study group

One hundred and thirteen participants were recruited for the study. Out of the group, 32 individuals did not complete the protocol. Data from the remaining 81 individuals were analyzed.

The study protocol was approved by The Bioethical Committee of the Medical University of Lodz (number: RNN/302/20/KE).

Following inclusion criteria were applied: informed consent to participate in both phases of the study (PSG and DS) evidenced by signing the appropriate form provided by the Department of Sleep Medicine and Metabolic Disorders, compliance with the study protocol, age above 18 and below 35, body mass index (BMI) 20–30 kg/m^2^.

Exclusion criteria were: pregnancy or breastfeeding, chronic diseases (inflammatory, endocrine, cardiopulmonary/renal insufficiency), radio or chemotherapy, neoplasms other than basal-cell carcinoma, surgery during the last 6 months, substance abuse, presence of sleep disorders, such as primary insomnia, obstructive sleep apnea, intercontinental flights 2 weeks before qualification or during the study, other psychiatric disorders (e.g., obsessive–compulsive disorder or bipolar disorder) based on the interview and questionnaires (Mood Disorder Questionnaire, Yale-Brown Obsessive–Compulsive Scale).

### Protocol

The study protocol involved 2 stages, PSG and DS. Participants were asked to lead a sleep diary 2 weeks prior to the PSG, according to the instructions provided by the researchers. This allowed for the collection of subjective data regarding total sleep time, time spent in bed, sleep quality, as well as number and duration of naps.

To perform a standard PSG examination, participants were admitted to the Department on the evening of the scheduled day. General physical examination and history taking were carried out by a physician shortly thereafter. PSG duration was set at 9 hours, from 10 PM to 7 AM.

PSG examination included a standard set of parameters: electroencephalography (EEG), electromyography (EMG), electrooculography (EOG), respiratory effort (chest and abdomen), airflow (oronasal thermistor placed underneath nose), electrocardiography, body position, blood oxygen saturation. Parameters were recorded with Alice 4, Phillips-Respironics (Monroeville, PA, USA). PSG recordings were interpreted by one researcher, to standardize results.

PSG findings were analyzed in concordance with the standards of the American Academy of Sleep Medicine; epoch length was set at 30 s [16]. Focus was placed on detecting signs of sleep disorders, e.g., obstructive sleep apnea or periodic limb movement disorder.

Participants with average total sleep time (based on data from sleep diary and PSG recording) below 5 h per day were excluded from further analysis.

DS was conducted between 2 and 4 weeks after the PSG and lasted ca. 24 h, beginning in the morning of the DS day. Analogously to PSG, participants were required to make a visit to the Department twice, in the evening and in the following morning. Enrolled individuals were allowed to keep themselves occupied throughout the night as long as it did not break the protocol.

Physical activity (PA) of study subjects was recorded with actigraphy (Actigraph GENEActive Original, ActivInsights Ltd.). Each participant was provided with the appropriate appliance during the evening visit to the Department. The results of actigraphy were presented as a gravity-subtracted sum of vector magnitudes. Actigraphy recordings were interpreted by one researcher to standardize the results.

Venous blood samples (9 ml), as well as questionnaires evaluating sleep quality, daytime sleepiness, and symptoms of insomnia and depression, were collected during each participant’s visit to the Department, in the evening before PSG, DS, and on the following morning.

Montgomery–Åsberg Depression Rating Scale (MADRS) is a scale evaluating depression severity, the prime focus of which is placed on mood rather than somatic aspects of this disorder. Ten items include, among others: tension, lassitude, and problems with concentration. A score above 7 is indicative of depression. Athens Insomnia scale (AIS) is an instrument used in the diagnostic process of insomnia [17, 18]. It contains 8 items, such as sleep continuity, sleep time, and distress; each is rated 0–3. A score above 5 is considered abnormal and suggestive of insomnia. Pittsburgh Sleep Quality Index (PSQI) is a questionnaire applied to the general assessment of sleep quality [19]. It covers a wide range of symptoms of inadequate sleep, like disrupted sleep continuity or impaired daily functioning. The cut-off point for poor sleep is 5. Epworth sleepiness scale (ESS) evaluates daytime sleepiness by inviting an individual to assess the probability of them falling asleep in 8 situations from daily life [20]. An outcome above 10 requires further investigation to detect the causes of excessive sleepiness.

Study subjects were divided into two groups based on changes in depression severity as measured with MADRS. MADRS score of Responders (RE) improved after DS or remained the same if the baseline was below 8, whereas that of non-responders (NR) worsened overnight.

### Molecular analysis

Isolation of RNA was conducted according to the Trizol method, with the use of TRIzol (Invitrogen). Evaluation of RNA concentration and RNA Integrity Number (RIN) was performed with a Nanodrop Colibri Microvolume Spectrometer (Titertek Berthold, Germany). Reverse Transcription Kit (SuperScript IV First-Strand Synthesis System, Thermo Fisher Scientific Inc., California, United States) was used to synthesize cDNA; the procedure was done in compliance with the manufacturer’s protocol.

Quantitative real-time polymerase chain reaction was performed to assess mRNA level of expression. Reaction mixture included: gene-specific probes (TaqMan assays for: BDNF, GDNF, NT3, NT4 reference gene: beta-actin). Assays underwent annealing at 60 °C for 60 s. For each sample, three reactions and a calculation of the cycle threshold (CT) were done. The obtained results were presented as ΔCT and analyzed with the Livak method [21].

### Biochemical analysis

The serum neurotrophin protein concentrations were assessed by appropriate ELISA kits (BDNF, GDNF, NT3, and NT4 FineTest; Wuhan, China). The procedure was conducted in accordance with the protocol provided by the manufacturer. Absorbance was measured at *λ* = 450 nm wavelength (BioTek 800 TS, Agilent Technologies, Santa Clara, CA, USA).

### Statistical analysis

Statistica 13.1 PL (StatSoft, Tulsa, OK, USA) was used in data analysis. The level of statistical significance was set at *p* < 0.05. Evaluation of distribution (normal or non-normal) was performed with the Shapiro–Wilk test. Data was presented as mean with standard deviation or median with interquartile range (IQR: first–third quartile) in case of normal and non-normal distribution, respectively. Independent parametric variables were analyzed with a student *t*-test, whereas the Wilcoxon or Mann–Whitney *U* test was applied for non-parametric dependent or independent ones, respectively. Correlations were calculated with Spearman’s correlation test.

## Results

Demographic data (age, sex, BMI, etc. of the study group (*n* = 81) were presented in Table 1; no statistically significant differences were noted between the groups (all *p* > 0.05). Depending on changes in MADRS score following DS, participants were divided into Responders (RE, *n* = 50) and Non-Responders (NR, *n* = 31), with the response rate as defined by assumed criteria being 61.7%.

**Table 1:** Baseline characteristics of study participants.

Parameter	Non-respondents	Respondents	*P*
*n*	31	49	–
Women, *n*, % Men, *n*, %	17, 54.8% 14, 45.2%	23, 46.9% 26, 53.1%	0.491
Age, median (IQR)	23 (22–26)	24 (22–26)	0.541
BMI, [kg/m^2^], median (IQR)	22.05 (19.49–23.78)	22.85 (22.04–24.72)	0.065
Smoking, *n*, %	3, 9.7%	6, 12.2%	0.723
Higher education, *n*, %	16, 51.6%	22, 44.9%	0.345
Total sleep time during the PSG night, [min], median (IQR)	400 (360–480.5)	430 (366–468.75)	0.987
Sleep latency during the PSG night, [min], median (IQR)	33.5 (17.5–51)	34.5 (23.75–57.75)	0.467
Sleep efficiency during the PSG night [%], median (IQR)	78.6 (71.7–89.5)	81 (69.25–85.65)	0.487

*BMI* body mass index, *IQR* interquartile range.

In comparison between the morning after PSG and DS, BDNF protein did not change (*p* = 0.731, *p* = 0.829 for RE and NR, respectively; Table 2.); *BDNF* mRNA, on the other hand, was reduced in all participants (*p* < 0.001) after DS. GDNF protein decreased in the NR group after DS compared to baseline (*p* = 0.030). RE, but not NR individuals, exhibited a decreased *GDNF* mRNA expression (*p* = 0.006, *p* = 0.554, respectively). NT3 protein increased after DS (*p* < 0.001) in all participants. *NT3* mRNA expression was reduced after DS in the RE but not NR (*p* = 0.009, *p* = 0.802, respectively). A post-DS increase in NT4 protein was observed in all study subjects (*p* < 0.001), while *NT4* mRNA expression was not impaired by DS (*p* = 0.392).

**Table 2:** Serum concentrations of neurotrophins and their respective gene expressions in the morning after polysomnographic examination and sleep deprivation.

	All			Respondents			Non-respondents		
	PSG morning *n*; median IQR	DS morning *n*; median IQR	*p*	PSG morning *n*; median IQR	DS morning *n*; median IQR	*p*	PSG morning *n*; median IQR	DS morning *n*; median IQR	*p*
BDNF protein	80; 4043,8 (1337.4–8247.0)	81; 3799.6 (1789.6–7972.7)	0.625	49; 3871.8 (1417.7–8810.4)	50; 3905.4 (1789.6–9068.9)	0.731	31; 4330.5 (947.3–7534.4)	31; 3607.3 (1588.1–7972.7)	0.829
GDNF protein	79; 66.5 (9.7–485.7)	80; 52.2 (14.0–496.6)	0.214	49; 45.7 (9.6–293.1)	50; 42.9 (16.6–384.0)	0.717	30; 98.0 (14.4–711.1)	30; 64.5 (7.8–665.3)	**0.030**
NT-3 protein	80; 102.5 (93.1–109.8)	81; 143.4 (131.5–165.7)	**<0.001**	49; 100.7 (92.8–109.7)	50; 143.3 (133.5–166.2)	**<0.001**	31; 104.2 (93.5–110.6)	31; 143.4 (131.0–162.3)	**<0.001**
NT-4 protein	80; 3273.0 (1627.6–5128.5)	81; 4868.7 (3048.5–6452.5)	**<0.001**	49; 3097.3 (1457.3–5764.0)	50; 5025.3 (2393.1–7246.6)	**0.001**	31; 3420.2 (2712.7–4889.0)	31; 4701.0 (3665.7–5945.2)	**0.013**
BDNF mRNA	75; 1117.3 (358.5–1438.9)	76; 165.6 (34.4–617.8)	**<0.001**	47; 1117.3 (380.2–1414.2)	47; 143.1 (33.7–628.5)	**<0.001**	28; 1119.2 (263.8–1508.5)	29; 179.9 (50.6–607.1)	**0.002**
GDNF mRNA	75; 768.4 (334.5–1117.3)	76; 344.5 (117.5–917.1)	**0.017**	47; 768.4 (430.8–1121.2)	47; 341.5 (103.3–864.5)	**0.006**	28; 795.0 (240.4–1117.3)	29; 407.5 (131.7–1042.5)	0.554
NT3 mRNA	75; 704.7 (308.9–1064.4)	75; 447.5 (115.0–820.7)	**0.040**	47; 704.7 (407.5–1064.4)	46; 471.2 (132.6–779.2)	**0.005**	28; 624.3 (244.59–1072.19)	29; 357.3 (115.0–858.6)	0.981
NT4 mRNA	75; 2848.1 (1136.8–6821.1)	76; 2220.4 (551.0–7298.0)	0.392	47; 2732.1 (1438.9–8282.1)	47; 1925.2 (491.4–8083.6)	0.397	28; 3759.2 (798.1–6109.5)	29; 2488.0 (823.6–5521.3)	0.699

*BDNF* brain-derived neurotrophic factor, *DS* deprivation of sleep, *GDNF* glial cell line-derived neurotrophic factor, *IQR* interquartile range, *NT-3* neurotrophin-3, *NT-4* neurotrophin-4, *PSG* polysomnography.

The bold values indicate statistical significance (*p* < 0.05).

On the between-group analysis, RE individuals showed a greater post-DS/post-PSG ratio of NT3 protein concentration than the NR (NR: 1.41, IQR: 1.29–1.51 vs RE: 1.47, IQR: 1.43–1.56, *p* = 0.018). In the case of other studied proteins and mRNA transcripts, the discussed ratio remained similar between the study groups.

PA was negatively correlated with post-DS *BDNF*, *GDNF*, and, *NT3* mRNA expression (*R* = −0.24, *p* = 0.040, *n* = 76; *R* = −0.30, *p* = 0.009, *n* = 76; *R* = −0.26, *p* = 0.023, *n* = 75, respectively), but not with *NT4* mRNA (*R* = −0.17, *p* = 0.148, *n* = 76) and with proteins (BDNF: *R* = 0.17, *p* = 0.120, *n* = 81; GDNF: *R* = −0.07, *p* = 0.565, *n* = 80; NT3: *R* = 0.11, *p* = 0.350, *n* = 81; NT4: *R* = −0.03, *p* = 0.801, *n* = 81).

## Discussion

Studies conducted in recent decades suggest that the beneficial influence of DS on depression might be caused by alterations in the synthesis of neurotrophins; literature on this subject covers primarily BDNF, while the role of other NTs remains elusive [22].

In the present study, BDNF protein concentration was not affected by DS. Results of projects on this subject conducted on individuals without comorbid psychiatric disorders, like depression, vary. Gorgulu et al. also did not note any changes in serum BDNF protein levels in their healthy controls [23]. However, in a later study on a smaller group (*n* = 17 in comparison to the previous *n* = 33) of younger individuals (19.82 ± 1.01 vs 35.00 ± 12.24 in the previous study), they observed an increase in serum BDNF protein concentration after 24 hours of DS, which was maintained after 36 hours [12]. Giaccobo et al. in a similar project on non-depressed individuals subjected to 24-h sleep deprivation, demonstrated that serum BDNF protein increased in the sleep-deprived group in comparison to the controls [24]. In contrast, Kuhn et al., in their study on young adults, observed a significant decrease in plasma BDNF protein; it must be noted that since BDNF is stored in the platelets, comparing protein concentration in plasma and serum might substantially differ, thus such comparisons are rather tentative [25]. It is also possible that the 24-h duration of DS applied in this study was not sufficient to note any significant changes in protein production. As already mentioned, acute sleep loss is famed for its rapid, albeit transient antidepressant effect in patients with depression, which has long been linked to BDNF increase [9]. Participants of the present study were not clinically depressed. However, the lack of differences between NR and RE groups regarding BDNF protein suggests the need for further investigation of the molecular background behind mood alterations induced by DS.

We noted that the expression of *BDNF* mRNA was significantly impaired in all participants. To the best of our knowledge, this is the first study to evaluate the expression of *BDNF* mRNA in peripheral blood leukocytes (PBL) under the conditions of DS; previous studies focused on region-dependent BDNF expression in the brain using animal models. For example, Fujihara et al. observed that in mice, *BDNF* mRNA was upregulated in the hippocampus but not in the brainstem or cerebellum in the wake of a sleepless night [26]. Due to the short *BDNF* mRNA half-life, alterations of this parameter could also more reliably reflect changes in BDNF production than measurements of protein concentration [27]. Stunted *BDNF* mRNA expression in the wake of DS could be a form of protection against an increase in proBDNF, which has opposite effects to its mature form, promoting apoptosis, synaptic elimination, etc. This model has certain restrictions. Since the vast majority of BDNF is produced in the central nervous system, alterations in the intensity of BDNF mRNA synthesis in the PBLs might not correlate with serum BDNF protein concentration.

Analysis of NT4 protein and its mRNA complements the findings discussed above, as those NTs are functionally redundant in mammals [28]. It was observed that NT4 serum protein increased in all participants after sleep loss compared to post-PSG outcomes. *NT4* mRNA production, on the other hand, remained at the same level regardless of response to DS. Based on such results, it could be inferred that DS propelled posttranscriptional processes, including the synthesis of mature NT4 from its pre-peptide. Post-DS rise in NT4 observed here could compensate for unaltered BDNF serum protein, which is usually seen in the aftermath of acute sleep deprivation [28].

In this study post DS serum GDNF protein was reduced only in the NR in comparison to their baseline values, whereas no significant changes were observed in the RE and when both groups were analyzed together. There is only one available study on the subject of the influence of DS on GDNF protein conducted by Gorgulu et al., where serum concentration of this NT, along with BDNF and VEGF, were elevated in all participants after 24 hours of lack of sleep [12]. The authors did not divide their study group based on mood response to DS [12]. Here, even though significant differences regarding this parameter in the between-group analysis were not present, the decrease in GDNF protein was observed only in subjects whose mood worsened after DS, suggesting that this NT bears a relation to emotional response to sleep loss. Differences in outcomes obtained by Gorgulu et al. and in our study could be to some extent ascribed to a smaller number (*n* = 17) of younger participants in the former, some of which would still be considered adolescents (aged 18–25, with a mean age of 19 years) [12]. *GDNF* mRNA expression was lower in all participants in the post-DS morning compared to the baseline; in the between-group analysis, we noted an opposite finding to that in the case of GDNF serum protein concentration, namely a decrease in *GDNF* mRNA in RE, but not NR. This suggests that sleep loss in RE stimulated the production of the mature NT from its pre-peptide, whereas in NR, posttranscriptional processes were disrupted.

As for NT3 protein, it was observed that its serum protein concentrations were significantly increased in the aftermath of DS. Moreover, the ratio of post-DS/post-PSG NT3 protein was higher in RE than in NR. The expression of *NT3* mRNA was lower in all participant regardless of their mood status after sleep loss. To the best of our knowledge, this is the first study to analyze those neurotrophins in the periphery under the influence of acute sleep loss. Hamatake et al., in their study on rat pups, observed that NT3 protein in the neocortex decreased after DS [29]. Studies have also shown that NT3 might have anxiolytic properties; in rats, blockade of BDNF/TrkB signaling, but not NT3/TrkC or NGF/TrkA was associated with alleviation of depressive or anxious behaviors in animals [30]. However, the molecular basis for such an effect is yet to be elucidated. Sheldrick et al. observed that NT3, as well as BDNF production, was elevated in different regions of the brains of individuals medicated for depression with selective serotonin reuptake inhibitors or tricyclic antidepressants compared to their counterparts whose depression was not managed pharmacologically [31].

What concerns PA, we found that it stunts the leukocyte expression of *BDNF*, *GDNF*, and *NT3* mRNA under the conditions of acute sleep loss. To the best of our knowledge, this is the first study assessing actigraphy scores in the context of NT productions during DS. Most studies on the interactions between PA and NTs focused on BDNF; available evidence shows that exercise might enhance its synthesis. Authors showed that rats with unlimited access to a running wheel exhibited an upregulated production of BDNF in different regions of the brain, hippocampus in particular [32]. In humans, acute exercise also upregulated both BDNF protein concentration in serum and gene expression in peripheral blood mononuclear cells; however, such effect was not present in the case of low-intensity training, thus showing that a certain threshold needs to be met to intensify BDNF production at least in the short term, which did not occur in the present study [33, 34]. As of 2023, there are no studies on the subject of PA and the expression of leukocyte-derived GDNF. Nevertheless, GDNF appears to have an important role in neuromuscular diseases, such as amyotrophic lateral sclerosis, where in animal models it could prevent the degeneration of motor neurons [35]. In a study on rats authors demonstrated that training might increase the GDNF protein concentration in the spinal cord [36]. Other researchers suggested that physical activity could be a good countermeasure attenuating the decrease in *GDNF* mRNA in muscles, preventing neurodegeneration [13]. Since sleep loss exerts a highly negative effect on physical performance, further investigation of alterations in *GDNF* mRNA expression and protein concentration in muscles following sleep deprivation would be desirable [37]. The analogous reaction of *NT3* mRNA to PA is not surprising since, similarly to GDNF, this NT is abundant in muscles, promoting their innervation [38]. Ying et al. also showed that exercise might upregulate the production of *NT3* transcript in muscles as well as the spinal cord of rats [39]. Future studies might consider analyzing the three discussed NTs together to determine how sleep and different types of PA influence the dynamics of their synthesis.

Albeit comprehensive, this study has several limitations. One of the most important is the relatively short DS duration, which was set here at 24 h. It is possible that applying total DS as well as analyzing blood samples at two time points: after 24 and 36 h of sleep loss, like did some other researchers, would give more insight into the dynamics of alterations in the synthesis of NTs under the influence of DS. In the present study analysis of immature forms of selected NTs as well as NGF was also omitted, as it could further convolute an already complex set of data, thus making it difficult to draw any conclusions. Actigraphy is considered to be a reliable method of monitoring one’s sleep/wake rhythm. However, it has its shortcomings, such as a lack of sufficient sensitivity to detect short naps.

To conclude, acute sleep loss influences the production of NTs from the level of transcription. PA under such circumstances seems to have a paradoxical effect on the mRNA expression of the discussed proteins, inhibiting their synthesis in leukocytes. GDNF, together with NT3, might play an important role in mood response to DS. Future studies on the subject of sleep deprivation might consider investigating the relationship between BDNF and NT4 in the context of their apparent redundancy, which implies a possibility of compensation. Projects exploring the balance between NT3 and BDNF in the periphery might also be validated, especially considering recent findings about the anxiolytic properties of the former.

## Data Availability

Data are available on request from the authors due to privacy restrictions.

## References

[1] Rahmani M, Rahmani F, Rezaei N. The brain-derived neurotrophic factor: missing link between sleep deprivation, insomnia, and depression. Neurochem Res. 2020;45:221–31. 31782101 10.1007/s11064-019-02914-1

[2] Vigers AJ, Amin DS, Talley-Farnham T, Gorski JA, Xu B, Jones KR, et al. Sustained expression of brain-derived neurotrophic factor is required for maintenance of dendritic spines and normal behavior. Neuroscience. 2012;212:1–18. 22542678 10.1016/j.neuroscience.2012.03.031 PMC4880414

[3] Sochal M, Ditmer M, Gabryelska A, Białasiewicz P. The role of brain-derived neurotrophic factor in immune-related diseases: a narrative review. J Clin Med. 2022;11:6023. 10.3390/jcm11206023 PMC9604720 36294343

[4] Schmitt K, Holsboer-Trachsler E, Eckert A. BDNF in sleep, insomnia, and sleep deprivation. Ann Med. 2016;48:42–51. 26758201 10.3109/07853890.2015.1131327

[5] Giese M, Unternährer E, Hüttig H, Beck J, Brand S, Calabrese P, et al. BDNF: an indicator of insomnia? Mol Psychiatry. 2014;19:151–2. 23399916 10.1038/mp.2013.10 PMC3903111

[6] Zhang P, Li YX, Zhang ZZ, Yang Y, Rao JX, Xia L, et al. Astroglial mechanisms underlying chronic insomnia disorder: a clinical study. Nat Sci Sleep. 2020;12:693–704. 33117005 10.2147/NSS.S263528 PMC7549496

[7] Tsybko AS, Ilchibaeva TV, Popova NK. Role of glial cell line-derived neurotrophic factor in the pathogenesis and treatment of mood disorders. Rev Neurosci. 2017;28:219–33. 28099138 10.1515/revneuro-2016-0063

[8] Grassi Zucconi G, Cipriani S, Balgkouranidou I, Scattoni R. One night’ sleep deprivation stimulates hippocampal neurogenesis. Brain Res Bull. 2006;69:375–81. 16624668 10.1016/j.brainresbull.2006.01.009

[9] Eckert A, Karen S, Beck J, Brand S, Hemmeter U, Hatzinger M, et al. The link between sleep, stress and BDNF. Eur Psychiatry. 2017;41:S282.

[10] Giese M, Beck J, Brand S, Muheim F, Hemmeter U, Hatzinger M, et al. Fast BDNF serum level increase and diurnal BDNF oscillations are associated with therapeutic response after partial sleep deprivation. J Psychiatr Res. 2014;59:1–7. 25258340 10.1016/j.jpsychires.2014.09.005

[11] Liu X, Li P, Ma X, Zhang J, Sun X, Luo X, et al. Association between plasma levels of BDNF and GDNF and the diagnosis, treatment response in first-episode MDD. J Affect Disord. 2022;315:190–7. 35908604 10.1016/j.jad.2022.07.041

[12] Gorgulu Y, Caliyurt O, Kose Cinar R, Sonmez MB. Acute sleep deprivation immediately increases serum GDNF, BDNF and VEGF levels in healthy subjects. Sleep Biol Rhythms. 2022;20:73–79. 38469072 10.1007/s41105-021-00341-w PMC10897642

[13] Vianney J-M, McCullough MJ, Gyorkos AM, Spitsbergen JM. Exercise-dependent regulation of glial cell line-derived neurotrophic factor (GDNF) expression in skeletal muscle and its importance for the neuromuscular system. Front Biol. 2013;8:101–8.

[14] Lippi G, Mattiuzzi C, Sanchis-Gomar F. Updated overview on interplay between physical exercise, neurotrophins, and cognitive function in humans. J Sport Health Sci. 2020;9:74–81. 31921482 10.1016/j.jshs.2019.07.012 PMC6943756

[15] Park YM, Lee BH. Alterations in serum BDNF and GDNF levels after 12 weeks of antidepressant treatment in female outpatients with major depressive disorder. Psychiatry Investig. 2018;15:818–23. 29945425 10.30773/pi.2018.03.31 PMC6111227

[16] Kapur VK, Auckley DH, Chowdhuri S, Kuhlmann DC, Mehra R, Ramar K, et al. Clinical practice guideline for diagnostic testing for adult obstructive sleep apnea: an american academy of sleep medicine clinical practice guideline. J Clin Sleep Med. 2017;13:479–504. 28162150 10.5664/jcsm.6506 PMC5337595

[17] Soldatos CR, Dikeos DG, Paparrigopoulos TJ. Athens Insomnia Scale: validation of an instrument based on ICD-10 criteria. J Psychosom Res. 2000;48:555–60. 11033374 10.1016/s0022-3999(00)00095-7

[18] Fornal-Pawłowska M, Wołyńczyk-Gmaj D, Szelenberger W. Validation of the Polish version of the Athens Insomnia Scale. Psychiatr Pol. 2011;45:211–21. 21714210

[19] Buysse DJ, Reynolds CF 3rd, Monk TH, Berman SR, Kupfer DJ. The Pittsburgh Sleep Quality Index: a new instrument for psychiatric practice and research. Psychiatry Res. 1989;28:193–213. 2748771 10.1016/0165-1781(89)90047-4

[20] Johns MW. A new method for measuring daytime sleepiness: the Epworth sleepiness scale. Sleep. 1991;14:540–5. 1798888 10.1093/sleep/14.6.540

[21] Schmittgen TD, Livak KJ. Analyzing real-time PCR data by the comparative CT method. Nat Protoc. 2008;3:1101–8. 18546601 10.1038/nprot.2008.73

[22] Boland EM, Rao H, Dinges DF, Smith RV, Goel N, Detre JA, et al. Meta-analysis of the antidepressant effects of acute sleep deprivation. J Clin Psychiatry. 2017;78:e1020–e1034. 28937707 10.4088/JCP.16r11332

[23] Gorgulu Y, Caliyurt O. Rapid antidepressant effects of sleep deprivation therapy correlates with serum BDNF changes in major depression. Brain Res Bull. 2009;80:158–62. 19576267 10.1016/j.brainresbull.2009.06.016

[24] Giacobbo BL, Correa MS, Vedovelli K, de Souza CE, Spitza LM, Goncalves L, et al. Could BDNF be involved in compensatory mechanisms to maintain cognitive performance despite acute sleep deprivation? An exploratory study. Int J Psychophysiol. 2016;99:96–102. 26602839 10.1016/j.ijpsycho.2015.11.008

[25] Kuhn M, Wolf E, Maier JG, Mainberger F, Feige B, Schmid H, et al. Sleep recalibrates homeostatic and associative synaptic plasticity in the human cortex. Nat Commun. 2016;7:12455. 27551934 10.1038/ncomms12455 PMC4996971

[26] Fujihara H, Sei H, Morita Y, Ueta Y, Morita K. Short-term sleep disturbance enhances brain-derived neurotrophic factor gene expression in rat hippocampus by acting as internal stressor. J Mol Neurosci. 2003;21:223–32. 14645989 10.1385/jmn:21:3:223

[27] Gass P, Hellweg R. Peripheral brain-derived neurotrophic factor (BDNF) as a biomarker for affective disorders? Int J Neuropsychopharmacol. 2010;13:1–4. 19995480 10.1017/S1461145709991039

[28] Bothwell M. NGF, BDNF, NT3, and NT4. Handb Exp Pharm. 2014;220:3–15. 10.1007/978-3-642-45106-5_1 24668467

[29] Hamatake M, Miyazaki N, Sudo K, Matsuda M, Sadakata T, Furuya A, et al. Phase advance of the light-dark cycle perturbs diurnal rhythms of brain-derived neurotrophic factor and neurotrophin-3 protein levels, which reduces synaptophysin-positive presynaptic terminals in the cortex of juvenile rats. J Biol Chem. 2011;286:21478–87. 21527636 10.1074/jbc.M110.195859 PMC3122207

[30] de Miranda AS, de Barros J, Teixeira AL. Is neurotrophin-3 (NT-3): a potential therapeutic target for depression and anxiety? Expert Opin Ther Targets. 2020;24:1225–38. 33141605 10.1080/14728222.2020.1846720

[31] Sheldrick A, Camara S, Ilieva M, Riederer P, Michel TM. Brain-derived neurotrophic factor (BDNF) and neurotrophin 3 (NT3) levels in post-mortem brain tissue from patients with depression compared to healthy individuals—a proof of concept study. Eur Psychiatry. 2017;46:65–71. 29102768 10.1016/j.eurpsy.2017.06.009

[32] Neeper SA, Gómez-Pinilla F, Choi J, Cotman CW. Physical activity increases mRNA for brain-derived neurotrophic factor and nerve growth factor in rat brain. Brain Res. 1996;726:49–56. 8836544

[33] Ferris LT, Williams JS, Shen CL. The effect of acute exercise on serum brain-derived neurotrophic factor levels and cognitive function. Med Sci Sports Exerc. 2007;39:728–34. 17414812 10.1249/mss.0b013e31802f04c7

[34] Brunelli A, Dimauro I, Sgrò P, Emerenziani GP, Magi F, Baldari C, et al. Acute exercise modulates BDNF and pro-BDNF protein content in immune cells. Med Sci Sports Exerc. 2012;44:1871–80. 22543740 10.1249/MSS.0b013e31825ab69b

[35] Morel L, Domingues O, Zimmer J, Michel T. Revisiting the role of neurotrophic factors in inflammation. Cells. 2020;9:865. 32252363 10.3390/cells9040865 PMC7226825

[36] McCullough MJ, Gyorkos AM, Spitsbergen JM. Short-term exercise increases GDNF protein levels in the spinal cord of young and old rats. Neuroscience. 2013;240:258–68. 23500094 10.1016/j.neuroscience.2013.02.063 PMC3637874

[37] Craven J, McCartney D, Desbrow B, Sabapathy S, Bellinger P, Roberts L, et al. Effects of acute sleep loss on physical performance: a systematic and meta-analytical review. Sports Med. 2022;52:2669–90. 35708888 10.1007/s40279-022-01706-y PMC9584849

[38] Sahib S, Sharma A, Menon PK, Muresanu DF, Nozari A, Lafuente JV, et al. Chapter 9—cerebrolysin enhances spinal cord conduction and reduces blood-spinal cord barrier breakdown, edema formation, immediate early gene expression and cord pathology after injury. In: Sharma HS, Sharma A (eds). Progress in Brain Research, 258. Elsevier, 2020, pp 397–438. 10.1016/bs.pbr.2020.09.012 33223040

[39] Ying Z, Roy RR, Edgerton VR, Gómez-Pinilla F. Voluntary exercise increases neurotrophin-3 and its receptor TrkC in the spinal cord. Brain Res. 2003;987:93–99. 14499950 10.1016/s0006-8993(03)03258-x

