# Correction: Interactions between neurotrophins, mood, and physical activity under the conditions of sleep deprivation

**DOI:** 10.1038/s41398-025-03462-9

**Published:** 2025-07-01

**Authors:** Marcin Sochal, Marta Ditmer, Agata Binienda, Aleksandra Tarasiuk, Piotr Białasiewicz, Szymon Turkiewicz, Filip Franciszek Karuga, Fichna Jakub, Agata Gabryelska

**Affiliations:** 1 Department of Sleep Medicine and Metabolic Disorders, Medical University of Lodz, Lodz, Poland; 2 Department of Biochemistry, Medical University of Lodz, Lodz, Poland

**Keywords:** Scientific community, Clinical genetics

Correction to: *Translational Psychiatry* 10.1038/s41398-024-02871-6, published online 22 March 2024

In the published article, there was an error in Table 1 as published. Group sizes and nominal variables (Women/Men, Smoking, Higher Education) were entered incorrectly. The corrected Table 1 appears below.

**Table 1:** Baseline characteristics of study participants.

Parameter	Non-Respondents	Respondents	P
n	31	49	–
Women, n, %	17, 54.8 %	23, 46.9 %	0.491
Men, n, %	14, 45.2 %	26, 53.1 %	
Age, median (IQR)	23 (22–26)	24 (22–26)	0.541
BMI, [kg/m^2^], median (IQR)	22.05 (19.49–23.78)	22.85 (22.04–24.72)	0.065
Smoking, n, %	3, 9.7 %	6, 12.2 %	0.723
Higher Education, n, %	16, 51.6 %	22, 44.9 %	0.345
Total Sleep Time during the PSG night, [min], median (IQR)	400 (360–480.5)	430 (366–468.75)	0.987
Sleep Latency during the PSG night, [min], median (IQR)	33.5 (17.5–51)	34.5 (23.75–57.75)	0.467
Sleep efficiency during the PSG night [%], median (IQR)	78.6 (71.7–89.5)	81 (69.25–85.65)	0.487

Body mass index (*BMI*), Interquartile range (*IQR*).

The authors apologize for this error and state that this does not change the scientific conclusions of the article in any way. The original article has been updated.The original article has been corrected.

